# Cervical cancer screening coverage, management of squamous intraepithelial lesions and related costs in France

**DOI:** 10.1371/journal.pone.0228660

**Published:** 2020-02-13

**Authors:** Yann de Rycke, Florence Tubach, Alexandre Lafourcade, Sylvie Guillo, Marie Dalichampt, André Dahlab, Xavier Bresse, Mathieu Uhart, Christine Bergeron, Hélène Borne, Charlotte Cancalon, Audrey Lajoinie, Stève Bénard

**Affiliations:** 1 Sorbonne Université, INSERM, Institut Pierre Louis d’Epidémiologie et de Santé Publique, AP-HP, Sorbonne Université, Hôpital Pitié Salpêtrière, Département de Santé Publique, Centre de Pharmacoépidémiologie (Cephepi), Unité de Recherche Clinique PSL-CFX, Paris, France; 2 Biostatistics consultant, Nantes, France; 3 Sanofi Pasteur MSD, Lyon, France; 4 MSD Vaccins, Lyon, France; 5 Laboratoire Cerba, Cergy-Pontoise, France; 6 Gynecologist, Paris, France; 7 stève consultants, Oullins, France; University of Vermont Larner College of Medicine, UNITED STATES

## Abstract

Until 2018, cervical cancer screening in France was an unorganized individual screening, with the exception of some pilot programs in some territories. We aimed to assess, before the implementation of organized cervical cancer screening and human papillomavirus (HPV) nonavalent vaccine introduction in the vaccination schedule in 2018, (i) the individual cervical cancer screening coverage, (ii) the management of squamous intraepithelial lesions (SIL) and (iii) the related costs. We used the *Système National des Données de Santé* (SNDS) (Echantillon Généraliste de Bénéficiaires [EGB] and Programme de Médicalisation des systèmes d’information [PMSI]) to assess the cervical screening coverage rate in France between January 1^st^, 2012 and December 31^st^, 2014, and to describe diagnostic investigations and therapeutic management of SIL in 2013. After extrapolation to the general population, a total of 10,847,814 women underwent at least one smear test over the 3-year study period, corresponding to a coverage rate of 52.4% of the women aged 25 to 64 included. In 2013, 126,095 women underwent HPV test, 327,444 women underwent colposcopy, and 9,653 underwent endocervical curettage; 31,863 had conization and 12,162 had laser ablation. Besides, 34,067 women experienced hospital stays related to management of SIL; 25,368 (74.5%) had high-grade lesions (HSIL) and 7,388 (21.7%) low-grade lesions (LSIL). Conization was the most frequent in-hospital therapeutic procedure: 89.5% (22,704) of women with an in-hospital procedure for HSIL and 64.7% (4,781) for LSIL. Mean cost of smear test, colposcopy and HPV tests were around 50€. Total cost for hospital stays in 2013 was estimated at M41€, or a mean cost of 1,211€ per woman; 76% were due to stays with HSIL. This study highlights the low coverage rate of individual cervical cancer screening and a high burden related to SIL management.

## Introduction

Cervical cancer is the fourth most frequent cancer in women worldwide for both incidence and mortality, with an estimated 570,000 cases and 311,000 deaths in 2018 [1]. In France, the projections from *Santé Publique France* and the French National Cancer Institute in 2017 were 2,840 estimated incident cases, corresponding to an incident rate of 6.0 per 100,000 persons per year, and 1,080 deaths, corresponding to a mortality rate of 1.7 per 100,000 [2].

It is now universally accepted that almost all cervical cancers are due to infection with human papillomavirus (HPV) [3,4]. HPV is the most common viral infection of the reproductive tract; most sexually active women and men will be infected during their life [4]. When an infection with high-risk HPV persists, it may lead to precancerous lesions either low grade squamous intraepithelial lesions (LSIL) for type 1 cervical intraepithelial neoplasia (CIN 1) or high grade squamous intraepithelial lesions (HSIL) for CIN 2 and 3. These precancerous lesions may progress to a cervical cancer within 10 to 20 years of evolution. To date, a total of 13 HPV genotypes have been classified as cervical carcinogens by the International Agency for Research on Cancer (IARC) [5]. HPV genotypes 16 and 18 would be responsible for about 50% of HSIL [6,7] (*i*.*e*. CIN 2 and 3) and about 70% of cervical cancers [8].

Advances in knowledge on pathological process and characterization of detectable HSIL have made cervical cancer an ideal candidate for prevention program, with an expected positive public health impact [9,10]. HPV vaccination and cervical smear test are the best ways to prevent cervical cancer. The prophylactic vaccination is part of primary prevention and the last vaccine, against 9 types of HPV, was included in the 2018 vaccination schedule in France [11,12]. The vaccination has been recommended for all girls aged 11 to 14 years, and with a possible catch-up for girls aged 15 to 19 years not vaccinated. Screening for cervical cancer is part of secondary prevention. It is based on a cervical smear test called “pap test”. The smear is taken by gynaecologist or midwife and less often by a general practitioner or lab biologist, in a simple and painless way. From the cervical smear, a cytological examination is performed and consists of a morphological analysis of cervical cells to detect early the presence of abnormal and precancerous cells that can develop into cancerous lesions [13] [14]. The HPV test could also be performed. It is a molecular detection method that allows the detection of nucleic acids in high-risk HPV genotypes [14]. Until 2018, cervical cancer screening in France was an unorganized individual screening, with the exception of some pilot programs in some territories [15]. The national organized screening program was one of the major actions of the 2014–2019 cancer plan [16], and since the decree of May 4, 2018, organized cervical cancer screening is part of health programmes (Article L. 1411–6 of the Public Health Code), which already included the organized breast and colorectal cancer screening programmes [17]. The organized cervical cancer screening is recommended for all sexually active women aged 25 to 65 years, every three years, after two negative smears taken one year apart [18]. The smear is covered by French health insurance, and as part of organized screening programmes is free of charge for women with very low incomes [19].

In addition to the cervical smear and HPV tests, other confirmatory diagnostic tools are used as biopsy, colposcopy to identify abnormalities in the mucous membrane of the cervix and to specify its topography, and curettage to research a glandular or squamous endocervical lesion [20]. Therapeutic management can be based on surgery, which consists of removing a fragment of the cervix (conization or partial hysterectomy) or the entire uterus (hysterectomy), or based on treatment by chemotherapy and radiotherapy [21].

We aimed to assess, before the implementation of organized cervical cancer screening and HPV nonavalent vaccine introduction in the vaccination schedule in 2018, (i) individual cervical cancer screening coverage, (ii) the management of SIL and (iii) associated costs, using the French health insurance databases.

## Materials and methods

### Data source

We used claims data from the *Système National des Données de Santé* (SNDS), more specifically the *Echantillon Généraliste de Bénéficiaires* (EGB), a 1/97^th^ random sample of the French health Insurance database that contains data for 670,000 beneficiaries [22]. The EGB is an anonymized reimbursement database built by a random selection of individual identification numbers, representative of the French population by age and by sex. It notably contains anonymised individual data on patient’s sociodemographic characteristics, outpatient medical and paramedical care, reimbursed drugs delivered by community pharmacies, laboratory tests, inpatient healthcare, related expenditures, outpatient medical procedures (including SIL diagnosis, investigations and therapeutic management), and, if applicable, patient’s date of death.

We also used another database from the SNDS, the national hospital discharge summary database to assess hospital activity (*Programme de médicalisation des systèmes d’information*, PMSI). The PMSI database provided information on hospitalized patients, notably demographic (*e*.*g*. sex, age, gender, department and region of residence) and medical data (*e*.*g*. dates of start and end of stays, length of stay, reasons for hospital admission, medical unit(s) of stays, medical procedures performed during the stay, costly drugs dispensed during the stay, and, where applicable, hospital inpatient date of death). The PMSI database also contains costs for each hospital stay [23]. Discharge diagnoses are coded using the International Classification of Diseases (ICD-10) [24]. [25–27].

### Study design

#### Cervical cancer screening coverage

A cross-sectional analysis was conducted to identify all smear tests performed in France between January 1^st^, 2012 and December 31^st^, 2014 among all-age women including children. This study period was defined considering the 3 year-interval recommended between two smear test [28], allowing the estimation of population coverage. It included the most recent data at the date of the analyses.

#### Annual management of squamous intraepithelial lesions (SIL): diagnosis, therapeutic procedures and hospital stays related to SIL

A cross-sectional analysis was performed to describe SIL diagnosis investigations, therapeutic management and hospital stays related to SIL from January 1^st^, 2013 to December 31^st^, 2013 among all-age women including children.

### Data collection

Procedures of interest were identified: smear tests, diagnosis investigations, therapeutic management (performed in the community, outpatient visit or in-hospital management). For each procedure, patient’s age and cost of the procedure were extracted. For each hospital stays of interest (i.e. related to SIL management), we collected patient’s age, severity of SIL (low, high or undetermined-grade), type of ward for the stay (medical or surgical), type of hospitalisation (day or conventional hospitalisation), and cost of the stay.

#### Cervical cancer screening coverage

Smear tests performed during outpatient visits (either in the community or at hospital/clinic setting) between January 1^st^, 2012 and December 31^st^, 2014 were identified in the EGB database using standard codes of medical procedures (CCAM coding system), healthcare professional codes (NAGP coding system) and laboratory tests (NABM coding system).

Selected procedures included both cervical cell sampling and acts related to cytology exams. Codes for cervical cell sampling included acts performed by gynaecologists or generalist practitioners (GP; CCAM code JKHD001 in the community; CCAM code JKHD001 and NGAP code K-3 for outpatient visits), by midwifes (NGAP codes SF-3,6 for 2012–2013 and SF-4,1 for 2014), or by laboratories (NABM code 9053). Procedures related to cytology exams are processed by laboratories (NABM code 0013; CCAM codes JKQP001 and JKQP008 before March 2014, and JKQX001, JKQX008, JKQX015 and JKQX027 after March 2014).

Smear tests were accounted on year N (i) if a cervical cell sampling and a cytology exam were performed in a timeframe shorter than 30 days, or (ii) in the absence of any cell sampling on year N, if a cytology exam was performed between February 1^st^, and December 31^st^ for the year studied (January has not been considered in order not to take into account cell samplings performed in December year N-1), or (iii) in the absence of any cytology exam on year N, if a cervical cell sampling was performed during the month of December of year N (to take into account cell sampling whose cytology exam can be performed over the following year).

#### Annual management of squamous intraepithelial lesions (SIL): Diagnosis and therapeutic procedures and hospital stays related to SIL

Diagnosis investigations, therapeutic management and hospital stays related to SIL and occurring in 2013 were identified using CCAM and NABM codes for procedures, and ICD-10 codes for hospital discharge diagnosis.

Diagnosis investigations included (i) tests for carcinogenic HPV processed by laboratories (NABM codes 0024 and 4127; CCAM code ZZQP173); and procedures performed by gynaecologists or GP, (ii) colposcopies (CCAM code JLQE002), (iii) biopsies (CCAM code JKHA002) and (iv) cervical curettage (CCAM code JKGD003).

Therapeutic management comprised all SIL specific cervical surgical procedures exclusively performed by gynaecologists or GP. We extracted (i) laser-free destruction of SIL lesions, including cryotherapy and cold coagulation (CCAM code JKND004), (ii) laser ablation (CCAM codes JKND003 and JKND002), (iii) cold knife or laser conization (CCAM code JFKA031), (iv) large loop excision of the transformation zone (LLETZ; CCAM codes JKFD002 and JKFE003), and (v) surgery, including trachelectomy and colpotrachelectomy (CCAM codes JKFA008, JKFA009, JKFA011, JKFA019 and JKFA030).

Women hospitalized for SIL or carcinoma *in situ* as principal, secondary or associated diagnosis were identified through the selection of any hospital stay with ICD-10 diagnosis code N87* (Dysplasia of cervix uteri (low, high or undetermined-grade)) or D06* (Carcinoma *in situ* of cervix uteri; Table 1). Stays with ICD-10 code for cervical cancer (C53) were excluded from the analysis, as the study focused on SIL lesions. Stays with diagnosis codes of interest were excluded if they were recorded for a male patient. Lastly, stays without any procedure code related to the diagnosis or treatment of SIL were reviewed using all diagnoses and procedures coded during the stay in order to select only those compatible with SIL management.

**Table 1:** List of ICD-10 codes selected for the identification of hospital stays with CIN or carcinoma *in situ* as principal, secondary or associated diagnosis.

Pathology	ICD-10 code	Description
**Dysplasia of cervix uteri**	**N87**	**Dysplasia of cervix uteri**
	*N870*	*Mild cervical dysplasia* *Cervical intraepithelial neoplasia [CIN]*, *grade I*
	*N871*	*Moderate cervical dysplasia* *Cervical intraepithelial neoplasia [CIN]*, *grade II*
	*N872*	*Severe cervical dysplasia*, *not elsewhere classified* *Severe cervical dysplasia NOS* *Excl*.: *cervical intraepithelial neoplasia [CIN]*, *grade III*, *with or without mention of severe dysplasia*
	*N879*	*Dysplasia of cervix uteri*, *unspecified*
**Carcinoma in situ of cervix uteri**	**D06**	**Carcinoma in situ of cervix uteri** **Incl.: cervical intraepithelial neoplasia [CIN], grade III, with or without mention of severe dysplasia** **Excl.: melanoma in situ of cervix (D03.5), severe dysplasia of cervix NOS (N87.2)**
	*D060*	*Carcinoma in situ of endocervix*
	*D061*	*Carcinoma in situ of exocervix*
	*D067*	*Carcinoma in situ of other parts of cervix*
	*D069*	*Carcinoma in situ of cervix*, *unspecified*

NOS, not otherwise specified

### Statistical analysis

Since the EGB database is a random sample of the French National Health Insurance, results presented in this article have been extrapolated to the overall French female population. The extrapolation coefficients from EGB to the general population of women corresponded to the ratio of the number of women in the EGB in 2012 reported to the female French population as of January 1^st^, 2013 by 5-year age class, and are presented in S1 Table. The number of women in the general population was obtained from the *Institut national de la statistique et des études économiques [INSEE]*.

Quantitative data are expressed as means and standard deviations or medians and interquartile range (IQR) for non-normally distributed variables. Categorical data are described using numbers and percentages.

Total number of smear tests and number of women with at least one smear test were calculated for the whole population of all age women. The cervical cancer screening coverage rate was then calculated (i) for all-age women, and (ii) for women aged between 25 and 64 included in order to estimate the adherence to French guidelines for cervical cancer screening that recommend screening in this age stratum [28]. The rate was estimated per age group by calculating the proportion of women that underwent at least one smear test between January 1^st^, 2012 and December 31^st^, 2014 reported to the size of the French female population, on January 1^st^, 2013 [29].

Cervical cancer screening coverage was reported by patient’s age categories considering 5-year age groups, and components of disease management (diagnosis investigations, therapeutic management and hospital stays) were reported for all women. Hospital stays were presented by severity level of the CIN lesion. Stays recorded with different severity grades were classified according to the most severe.

An economic analysis has been performed determining costs related to smear test, diagnosis investigation, therapeutic management and hospital stays related to SIL, by item of expenditures, from a collective perspective including direct medical costs. Mean cost per patient for procedures performed during outpatient visits presented for reimbursement has been extracted. Inpatient healthcare costs were estimated per hospital stay, based on the French national Diagnosis Related Group (DRG). The DRG is determined, for a given stay, based on recorded discharge diagnosis and classifying procedures performed during the stay. Costs related to each DRG—*i*.*e*. that cover treatments (except expensive drugs), medical procedures, nursing and physician fees—is assessed on the basis of the annual national cost study (ENCC). Costs are reported in Euros (€), year 2016, with costs prior to 2016 being revalued according to a consumer price index—health data index (4011-E)—published by INSEE [30].

All analyses were performed using SAS^®^ V9.3 (SAS Institute Inc. Cary, NC, USA).

### Ethics

Study methods were validated by a multidisciplinary qualified independent scientific committee (ISC). This observational study used solely existing claims data (SNDS, including EGB and PMSI databases). According to the French legislation at the time of study implementation, process to access such administrative database did not involve submission to an ethics committee. Since the study used existing data from the SNDS, patients did not need to be personally informed of this study. Permanent access to the EGB database was granted for Inserm units according to French legislation, and permission to extract and use the PMSI data by the consultancy company was obtained from the National Commission on Informatics and Liberty (CNIL; authorization n° 1976885). The SNDS security repository is provided for by the law n°2016–41 of January 26^th^, 2016 of modernization of the French health system (Title VI of the Public Health Code) to ensure personal data confidentiality and integrity. The SNDS must not contain any directly identifying data. Any access to an SNDS dataset should only be open for a fixed period, in accordance with the period specified in the authorization granted by the CNIL or other conditions provided for by law, and for trained, qualified and authenticated users.

## Results

### Cervical cancer screening coverage (2012–2014)

Between January 1^st^, 2012 and December 31^st^, 2014, a total of 15,145,887 smear tests were performed in all-age women. The total number of smear tests performed per year was 5,099,325 in 2012, 5,097,960 in 2013, and 4,948,602 in 2014. This corresponds to a total number of 10,847,814 women with at least one smear test performed for the whole female population over the recommended 3-year interval, corresponding to 4,906,170–4,901,131 and 4,758,381 women in 2012, 2013 and 2014, respectively. Among women with at least one smear test over the study period, the mean number of smear tests per woman per year remained stable for the 3 years studied, at 1.40.

**Fig 1:**
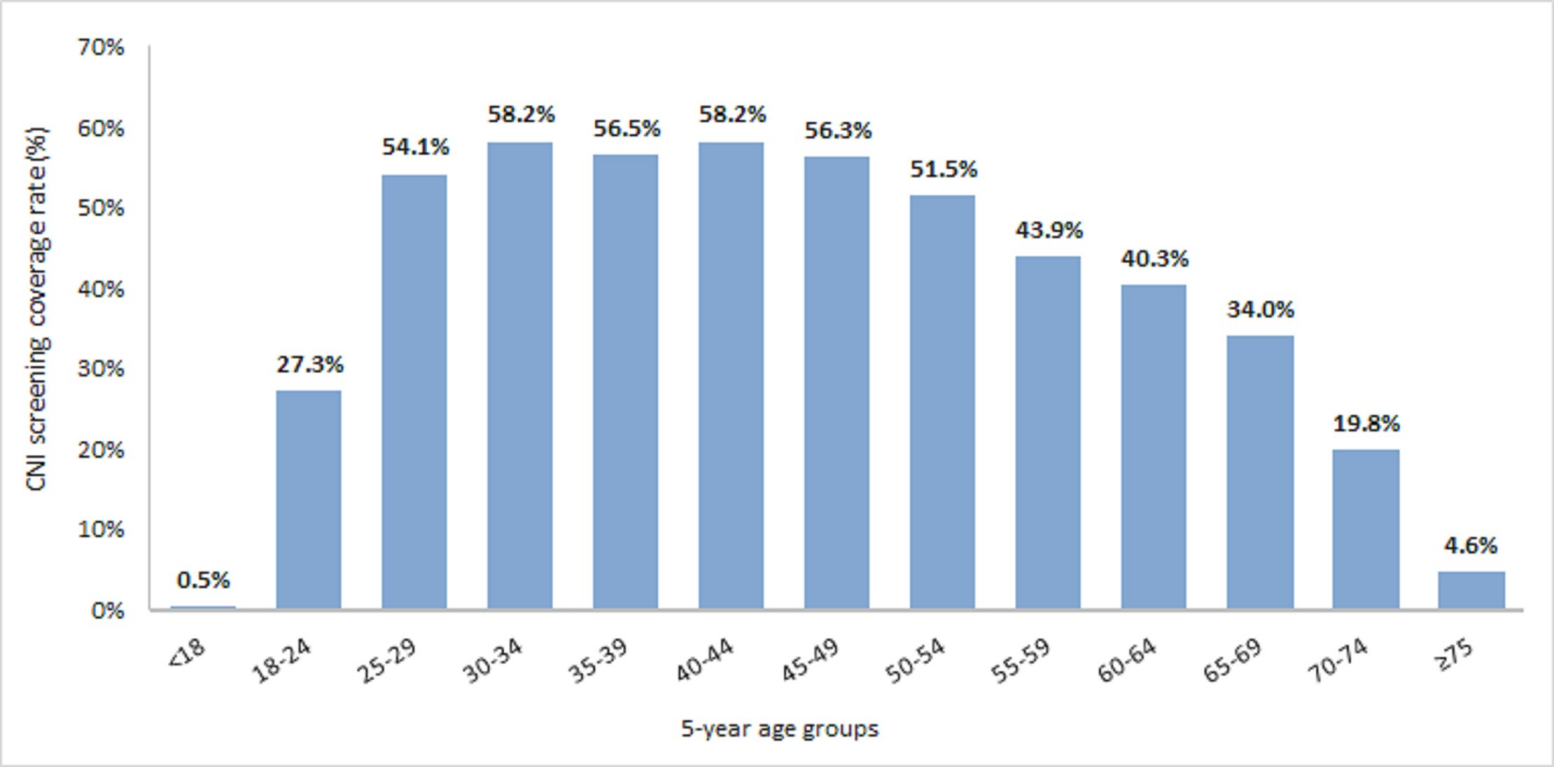
Cervical cancer screening coverage rate in France over 2012–2014 (between January 1st, 2012 and December 31st, 2014), i.e. proportion of women with at least one smear test performed within the 3-year period reported to the size of the French female.

The overall screening coverage rate in 25–64 years inclusive age group women over the 3-year study period was 52% of the women. It was 56 to 58% among the 30–49 age categories and decreased in older age groups to reach 40% among 60–64 years inclusive and 34% among 65–69 age group women (Fig 1).

### Annual management of squamous intraepithelial lesions (SIL): Diagnosis and therapeutic procedures and hospital stays related to SIL in 2013

Concerning diagnosis investigations, 131,172 HPV tests were performed in 2013, corresponding to 126,095 women with at least one HPV test over the study period of, accounting for 2.6% of women with at least one smear test over the year (Table 2). Over the same year, 327,444 women underwent at least one colposcopy with a total of 369,967 colposcopies. Only 45,779 biopsies have been identified in 2013, processed in 43,173 women. Lastly, curettage accounted for 9,653 procedures in 9,653 women.

**Table 2:** SIL management in 2013, all-age women in the general population*.

	Number of events	Number of women with at least one event
**Diagnosis investigation**		
*Smear tests*	5,097,960	4,901,131
*HPV test*	131,172	126,095
*Colposcopy*	369,967	327,444
*Biopsy*	45,779	43,173
*Curettage*	9,653	9,653
**Therapeutic management**		
Conization^**^	36,661	31,863
*Laser ablation*	15,162	12,162
Laser-free destruction^***^	13,737	12,561
*LLETZ*	12,456	11,221
**Hospital stays**		
*Hospital stays with diagnosis of SIL or carcinoma in situ*	35,555	34,067

* Results presented for the general population of women from the coefficients of extrapolation of EGB described in S1 Table.

** Laser or cold knife

*** Including cryotherapy and cold coagulation

Regarding SIL therapeutic management, for recommended procedures, 36,661 conizations and 15,162 laser ablations were performed in 2013; this corresponds to a number of 31,863 and 12,162 women who underwent at least one of those procedures, respectively (Table 2). Lastly, 13,737 laser-free destruction procedures and 12,456 LLETZ were performed in 2013.

In 2013, 34,067 women were hospitalized with a diagnosis of SIL (corresponding to 1.03 ‰ French women in 2013), for a total number of 35,555 hospital stays related to management of SIL (Table 2). Among those women, 25,368 (74.5%) had HSIL (CIN 2+), with a mean age of 38.6 ± 11.0 years old; they experienced a total of 26,760 stays, or a mean number per woman of 1.05 stays over the year (Table 3). Women with LSIL (CIN 1) accounted for 7,388 (21.7%), with a mean age of 40.3 ± 12.1 years old; they experienced a total of 7,455 stays, or 1.01 hospitalisation per woman. Lastly, 1,311 (3.8%) women had unspecified grade, with a mean age of 43.9 ± 12.7 years old; the total number of stays was 1,340, or a mean number of 1.02 stay per woman.

**Table 3:** Hospital stays related to management of SIL, all-age women in the general population*.

	HSIL	LSIL	Lesions of unknown grade	Total
**Number of women**	25,368	7,388	1,311	34,067
**Number of hospital stays**	26,760	7,455	1,340	35,555
	n (% of stays)	n (% of stays)	n (% of stays)	
**Nature of the stay**				
*Surgery*	26,131 (97.6)	7,261 (97.4)	1,132 (84.5)	34,524 (97.1)
*Medicine*	629 (2.4)	194 (2.6)	208 (15.5)	1,031 (2.9)
**Type of hospitalisation**				
*Day hospitalisation*	21,575 (80.6)	6,336 (85.0)	931 (69.5)	28,842 (81.1)
*Conventional hospitalisation*	5,185 (19.4)	1,119 (15.0)	409 (30.5)	6,713 (18.9)
	n (% of women)	n (% of women)	n (% of women)	
In-hospital procedures**				
Conization^***^	22,704 (89.5)	4,781 (64.7)	744 (56.8)	28,229 (82.9)
*Curettage*	2,480 (9.8)	561 (7.6)	67 (5.1)	3,108 (9.1)
*Laser ablation*	1,169 (4.6)	1,709 (23.1)	95 (7.2)	2,973 (8.7)
Surgery^****^	1,916 (7.6)	392 (5.3)	11 (0.8)	2,319 (6.8)
*Laser-free* destruction^*****^	108 (0.4)	184 (2.5)	11 (0.8)	303 (0.9)

*Results presented for the general population of women from the coefficients of extrapolation of EGB described in S1 Table.

** Only in-hospital procedures are accounted; both outpatients and inpatient procedures are accounted to assess the burden of the disease in Table 2. A same woman could have had more than one procedure.

** *Laser or cold knife

*** *Trachelectomy and colpotrachelectomy.

***** Including cryotherapy and cold coagulation

Surgical hospital stays represented the large majority of stays related to SIL management, regardless of the SIL grade (97.6% of all stays for high grade, 97.4% for low grade and 84.5% for undetermined; Table 3). Most of stays corresponded to day admissions (80.6% of all stays for HSIL, 85.0% for LSIL and 69.5% for undetermined; Table 3).

Regarding in-hospital management, most of women underwent a cervical conization during the stay, which was the most used therapeutic management for all SIL grade: 89.5% for women with HSIL, 64.7% for LSIL and 56.8% for undetermined (Table 3). Other major procedures for hospitalized SIL were diagnostic curettage, surgery, and laser ablations (Table 3).

### Economic analysis

**Fig 2:**
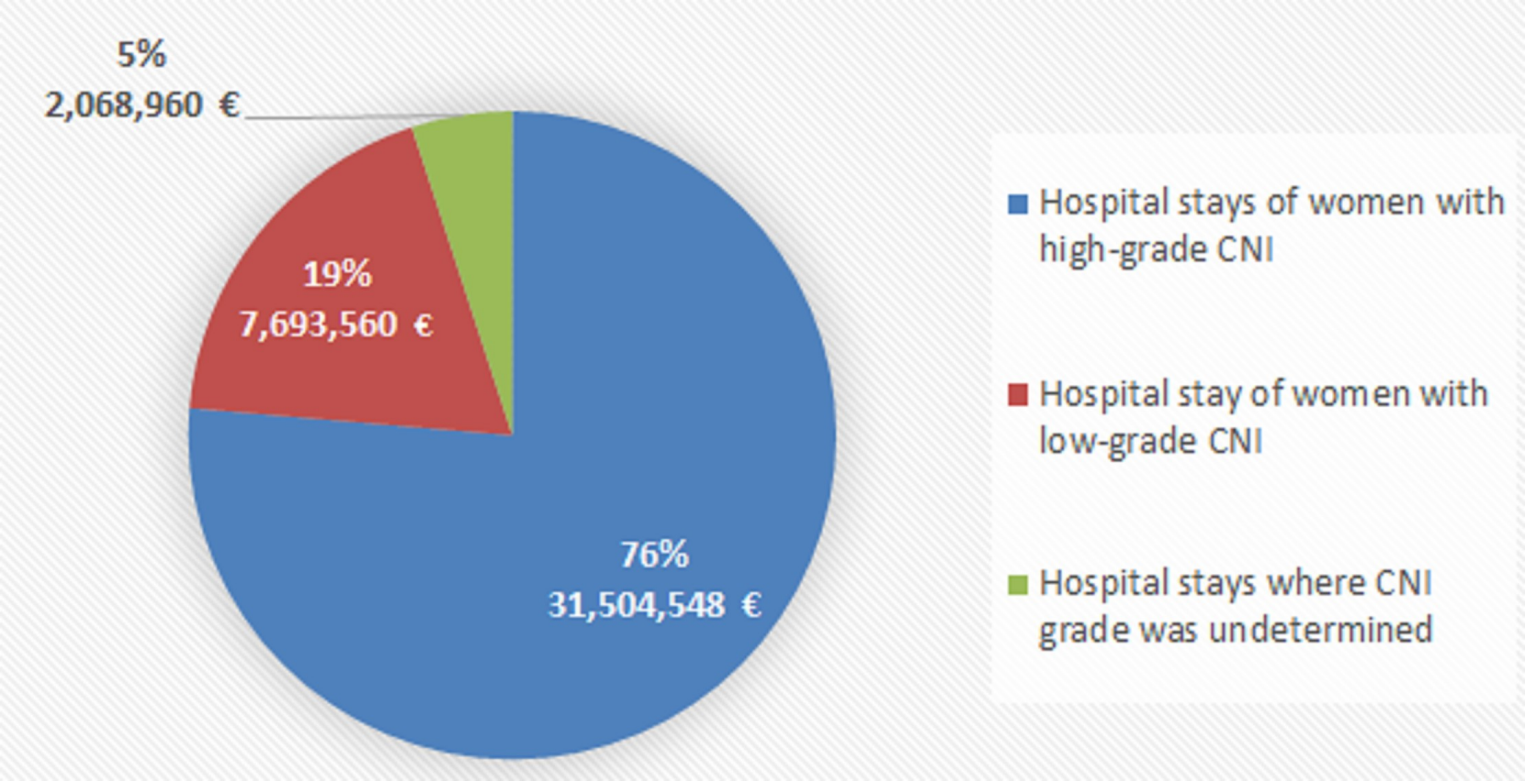
Distribution of costs arising from hospital stays related to CIN management in 2013, depending on CIN grade.

Mean cost per procedure for diagnosis and therapeutic procedures was based on outpatient and inpatient costs. The mean cost for a complete smear test (*i*.*e*. a cervical smear test associated with a cytological examination) was 56.5 ± 26.9 €, and the mean cost for HPV test was 41.1 ± 7.9 €. Concerning management related to SIL in hospital, the average cost per hospital stay for women with HSIL was 1,177.3 ± 1,046.9 €, corresponding to a total cost of 31,504,548 € for the total number of 26,760 stays (Fig 2). The mean cost per hospital stay for women with LSIL was 1,032.1 ± 812.0 €, or a total cost for the 7,455 stays of 7,694,305 €. Lastly, the average cost per hospital stay where SIL grade was undetermined was 1,544.0 ± 1,397.8 €, with a total cost of 2,068,960 € for the 1,340 stays. The total cost for hospital stays in 2013 was estimated at 41,267,068 € for the 34,067 women who were hospitalized with a diagnosis of SIL, or a mean cost of 1,211 € per woman. Seventy-six per cent of the total cost arisen from stays with HSIL diagnosis; stays with LSIL accounted for 19% of total costs.

## Discussion

This study, based on the French SNDS (EGB and PMSI databases), provides robust data on cervical cancer screening and on the burden of SIL lesions before the implementation of the organized cervical cancer screening and the introduction of the HPV non avalent vaccine. Indeed, health care consumption data recorded into this database presents the main advantage to be prospectively and exhaustively collected, independently of the study. The present study focused on outpatient and in-hospital diagnosis investigations and therapeutic management of SIL lesions; the management of potentially HPV-related cancers has already been assessed within a first observational study based on the French SNDS [31].

The screening coverage rate was estimated to be 52% in the French female population aged 25 to 65 years. The result is consistent with coverage rates measured in older French studies, ranging between 53% (2009–2011) and 62% (2010–2012) [32–37]. Although the targeted female population is mostly covered in France, coverage rate remains insufficient in regards of the objective in terms of cervical cancer screening coverage rate of 80% recommended by the French Cancer Plan 2014–2019 [38]. Cervical cancer screening coverage rate in France also seems to be below those of northern European countries [39]. On the other hand, women with at least one smear test within the 3-year of the study period had a mean number of 1.40 smear test per woman, suggesting an over screening. Similar results have been found in the French study conducted on the French health insurance databases, where the mean number of smear tests per women with at least one smear test was estimated at 1.5 in 25–64 years old women [40]. The 60–64 age group had the lowest coverage rate (40.3%) of the population targeted by the screening; this suggests the need of a strengthened screening policy for this age group, otherwise the lack of surveillance associated with population ageing could lead to an increase in number of cervical cancer in the future.

However, these findings have to be interpreted in light of study limitations. Notably, smear tests performed during hospital stays cannot be identified in the EGB database; while the proportion of smear tests performed during hospital stays has been estimated at 6.5% [41]. Thus, it is reasonable to believe that the screening rate observed in our study is slightly underestimated. Besides, the coding system does not allow us to distinguish screening smear tests from follow-up smear tests. Lastly, another argument supporting an underestimation of the number of smear tests is that cytology exam performed in January were not accounted as a smear test if no cervical cell sampling were recorded over the study period.

In France, HPV tests are only reimbursed in case of smear tests evocating ASC-US (Atypical Squamous Cells of Undetermined Significance) [42]. From the literature, ASC-US represented 2.5% of all HPV tests performed in 2008 in France; this is in accordance with our results showing that 131,172 HPV tests were performed in 2013, representing 2.6% of all smear tests [43]. The number of biopsies found using the CCAM code JKHA002 cannot be interpreted because the CCAM code for colposcopy (JLQE002) also includes an eventual biopsy. In addition, the codes for cervical biopsies (NGAP 004), for cervical curettage (NGAP 007) and conizations (NGAP 008) read in biological laboratories are non-specific and were therefore not used in this study, potentially leading to an underestimation. Conization–inclusion cold knife and laser conization—was performed in 31,863 women in 2013. The number of women having undergone destruction (n = 12,162) could have been slightly overestimated since the coding system for laser ablation also encompasses other lesions such as vulvar, vaginal, and perianal lesions. However, theses lesions are very sparse and can be considered as negligible compared with SIL [7,40,41]. Moreover, the number of laser-free destruction procedures and LLETZ (13,737 and 12,456, respectively) was not negligible. These procedures were not recommended for the management of SIL at the study period; but currently the use of LLETZ for carcinoma *in situ* and HSIL with a satisfactory colposcopy is recommended since the 2016 guidelines [42].

The analysis of hospital data allowed us to describe the in-hospital management of SIL. The total number of women hospitalized for a high grade (n = 25,368) or a low grade lesion (n = 7,388) is in accordance with previous data [7,43], accounting for respectively 8 and 2 women per 10,000 for the whole women population in France in 2013. This trend contrasts with those observed on other countries, such as Denmark, where high coverage rates for both cervical cancer screening and HPV vaccination, through a national HPV vaccination program, has been found to significantly decrease the number of SIL [7,44]. Among women hospitalised with diagnosis of HSIL, the proportion of patients that underwent conization (89.5%), surgery (9.8%) or laser ablation (4.6%) is in line with recommendations of French Health authorities [45]. HSIL should indeed always be treated and conization is the treatment of choice. To the opposite, treatment of low grade lesions is not systematic and hospital admission as well as conization are not systematically recommended. Mainly, from most recent recommendations from the French National Cancer Institute (*Institut National du Cancer*, INCa), treatment of LSIL lesions should only be considered for lesions that persisted after a 2-year monitoring period, with visit frequency depending on cytology results or HPV test results [42]. Our results indicate that 7,388 women were hospitalized with diagnosis a LSIL lesion in 2013, and 65% of them underwent conization. Similar findings have already been observed in a French study conducted on year 2004, where 6,637 hospital admission with LSIL diagnosis have been identified, and 3,693 conizations were performed [46]. Since conization may result in severe complications, especially in women of childbearing age, such as miscarriages in the second semester [47,48] or premature births [49,50], the risk/benefit balance of this procedure should be carefully assessed in each woman. Also, the organized cervical cancer screening program provides a homogenization of the management of abnormal smear tests to avoid excessive conizations and to reduce over-treatment [38]. Moreover, the HPV vaccine is a primary prevention measure that would prevent SIL, and the overtreatment and additional costs related with the treatment of these lesions. Surgical hospital stays represented the vast majority of stays related to SIL management while most of stays corresponded to day admissions. This result can be explained by a high ratio of conization performed as outpatient visit; thus most patients leave the hospital on the same day.

Mean costs of the diagnosis and treatment procedures were estimated based on costs presented to the reimbursement, as registered into the French Health Insurance databases. The total cost for hospital stays in 2013 was estimated at M41 €, or a mean cost of 1,211 € per woman; 76% were due to stays with HSIL diagnosis, and 19% to stays with LSIL.

Events of interest were identified through the SNDS, widely used and well-known tool for pharmaco-epidemiological and health economics studies [25]. Being aware of potential limits related to the use of these databases—mainly limited clinical details, limited validity of ICD coding -, we took special care to develop an optimized algorithm that properly identified all procedures related to SIL diagnosis and management. However, these results have to be interpreted in light of the limits of the study. Some procedures such as biopsy or LLETZ may indeed be under- or miscoded because of low financial valuation. However our results were shown to be consistent with figures reported by the French Health authorities and French epidemiological studies [28,41,43,49].

## Conclusion

This study highlights the low coverage rate of individual cervical cancer screening and the high burden of SIL, in terms of diagnostic investigations, therapeutic management and costs, in the French women population. These results must now be compared with those after implementation of the organized cervical cancer screening.

## Supporting information

S1 Table Coefficient of extrapolation of EGB to the general population in women. (DOCX)
