# Supplementary material for: Cervical cancer screening coverage, management of squamous intraepithelial lesions and related costs in France
Source: PLoS One. 2020 Feb 13;15(2):e0228660. doi: 10.1371/journal.pone.0228660 (PMC7018036; doi:10.1371/journal.pone.0228660)
Supplement: S1 Table — (DOCX) [file pone.0228660.s001.docx]

Supplementary table 1 - Coefficient of extrapolation of EGB to the general population in women

| **Age range** | **Coefficient of extrapolation in women** |
| --- | --- |
| **< 18 ans** | 110,58 |
| **18-24 ans** | 126,50 |
| **25-29 ans** | 103,73 |
| **30-34 ans** | 102,66 |
| **35-39 ans** | 105,13 |
| **40-44 ans** | 107,77 |
| **45-49 ans** | 109,88 |
| **50-54 ans** | 112,26 |
| **55-59 ans** | 115,10 |
| **60-64 ans** | 117,12 |
| **65-69 ans** | 117,85 |
| **70-74 ans** | 117,28 |
| **≥75 ans** | 105,01 |
| **Total** | 110,85 |
